# Optimization of Resistance Spot Welding with Inserted Strips via FEM and Response Surface Methodology

**DOI:** 10.3390/ma14237489

**Published:** 2021-12-06

**Authors:** Yangyang Zhao, Wurong Wang, Xicheng Wei

**Affiliations:** 1 State Key Laboratory of Advanced Special Steel, Shanghai University, 333 Nanchen Rd., Shanghai 200444, China; wrwang@shu.edu.cn (W.W.); wxc1028@shu.edu.cn (X.W.); 2 School of Materials Science and Engineering, Shanghai University, 333 Nanchen Rd., Shanghai 200444, China

**Keywords:** resistance spot welding, inserted strips, heat transfer, electrode tip temperature, finite element modeling, response surface methodology

## Abstract

Resistance spot welding (RSW) with inserted strips, a recent variant of traditional RSW, was usually adopted in joining thin gage steels to lower the temperature developed at the electrode surface and to extend electrode life. In order to understand the influencing mechanism how the inserted strips affect the heat transfer behavior and to optimize the selection of suitable strips, an approach integrated with FEM and response surface methodology (RSM) was employed. FEM results showed that the inserted strips would not only lead to earlier initiation of weld and bigger weld size in both diameter and thickness but also lower the electrode surface temperature. Based on FEM, uniform design and RSM were further employed to build a regression model between the strip properties (i.e., electrical/thermal conductivity, thickness) and the responses (i.e., electrode tip temperature, weld diameter, and temperature at strip/sheet interface). A graphical optimization was conducted to identify a preferable strip, and a Cu55Ni45 strip with a thickness of 0.12 mm was recommended for a 0.4 mm steel sheet.

## 1. Introduction

Facing the increasing pressure of the fuel crisis, vehicle light-weighting is an effective approach to reduce fuel consumption and emissions in the automotive industry. Reduction in weight is usually achieved by using lightweight materials (e.g., aluminum) or by optimization of structural design throughout the vehicle structures. For now, most of the vehicle outer panels are still made of mild steel with the thickness in the range of 0.65–0.8 mm [1]. Analyses indicated that if these panels can be substituted by advanced high strength steel sheets whose thicknesses are below 0.6 mm or even thinner, significant weight reduction could be achieved [1]. Moreover, the application of ultra-thin steels would be more economical than the use of aluminum [1].

However, resistance spot welding (RSW), as a commonly used welding method for vehicle assembly, is facing difficulties and limitations in the joining of thin gage (e.g., <0.6 mm) steels. One of the major challenges is that an extraordinary high temperature at the electrode surface can be developed, due to the fact that heat generated in the weld zone would transfer to the electrode surface more readily when the steel sheets become thinner. This excessively high temperature significantly accelerates the electrode degradation and eventually results in a reduction in electrode life by 40–60% compared to ordinary gage sheet [2]. Recently, a new variant of traditional RSW, RSW with metal strips inserted between the sheet and electrode, has been adopted to improve weld quality and extend electrode life in joining thin gage steels [3,4], aluminum [5,6,7], magnesium [8,9,10,11], and dissimilar metals [12,13,14,15]. The heat transfer behavior and temperature distribution pattern could be adjusted by altering the properties of the strips, and an improved weld quality and prolonged electrode life could be achieved if the inserted strips are well selected.

So far, the published studies of RSW with inserted strips mostly focused on exploring the possibility of using the inserted strips to join the hard-to-weld material combinations and comparing the joint quality between RSW with and without inserted strips. Understanding of how the inserted metal strips alters the heat generation and transfer in RSW is still limited. Due to the closed and transient nature of the RSW process, it is considerably difficult to measure the temperature history and distribution in the weld zone by experimental approaches. Zhao et al. [3,4] built an analytical model to qualitatively analyze the influence of strip properties on the electrode surface temperature and weld size. This analytical model insightfully pointed out the most crucial strip properties (i.e., thermal/electrical conductivity, thickness) on the heat transfer behavior, nevertheless, a quantitative calculation of the heat transfer and temperature distribution would further improve our understanding of the process of RSW with inserted strips and pave the road for process optimization.

Furthermore, RSW is considered as a multi-input multi-output process, and the introduction of inserted strips makes the welding process even more complicated. Therefore, it is difficult to analyze the effect of each input parameter by using the mono-factor analysis one by one. Response surface methodology (RSM) is a combination of mathematic and statistical techniques that is widely used for modeling and analyzing the complex problems in which the responses of interest are affected by several quantifiable factors and for finding the optimum of the responses of interest in many different research fields. It has been successfully adopted to optimize the process parameters to obtain desired joint properties [16,17,18]. Furthermore, Sohail employed RSM to investigate the effect of the eco-friendly finishing treatment parameters on the flame retardant properties and mechanical properties of cotton fabric and to obtain the optimal process parameter combination [19,20].

Therefore, this study aims to build a thorough understanding of the influential mechanism of the inserted metal strips on the heat transfer behavior during RSW of thin gage steels (i.e., thickness between 0.4–0.8 mm) and to optimize the selection of inserted strips using an approach integrated with FEM and RSM. Firstly, a finite element model coupling electrical, thermal, and mechanical analysis was constructed to simulate the heat transfer and temperature distribution in RSW with inserted strips. Then, uniform design and RSM was applied to establish the mathematical relationships between the input parameters (i.e., strip properties and welding parameters) and the responses (i.e., electrode tip temperature, weld diameter, and temperature at strip/sheet interface) based on the FEM. The effect of each input parameter on the responses was quantitatively analyzed, and the process window of feasible strip was also determined by using the overlay plot of responses. Finally, a preferable strip for a 0.4 mm steel sheet was selected based on graphical optimization of the response surfaces. Experiments were also carried out to validate the optimized result.

## 2. Finite Element Modeling

### 2.1. Model Construction

#### 2.1.1. Geometrical Model

**Figure 1:**
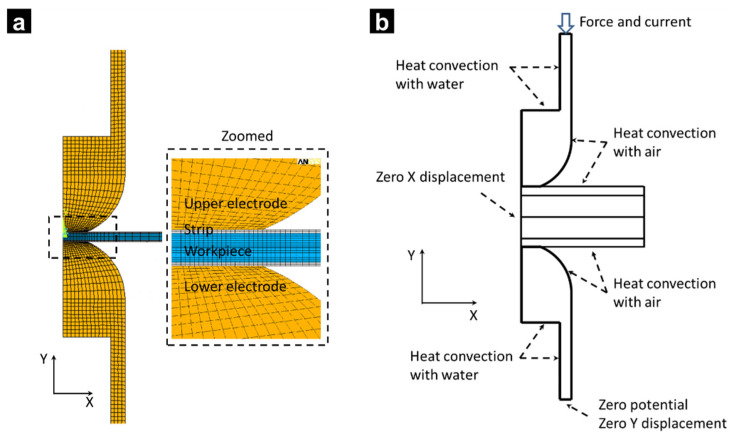
(**a**) Geometrical model and (**b**) boundary condition [4]. Reprinted with permission.

FEM was conducted using the module of Mechanical APDL in ANSYS 15.0. Considering the symmetry of RSW system, the FEM model was simplified to a 2D axial symmetric one. As shown in Figure 1a, four-node elements were used to model the electrode, strips, and steel sheets. Contact elements were inserted between the electrode/strip, strip/sheet, and sheet/sheet interfaces to model the contact phenomenon at interfaces. The mesh size was smaller at the center of weld zone, as shown in the enlarged area of Figure 1a.

The element type of electro-thermal analysis is PLANE67 and in the electro-thermal analysis, the element type of thermal-mechanical analysis is PLANE42. Both of them are 2D four-node elements, therefore, the software can automatically convert the element type in the iterative electro-thermal/thermal-mechanical calculation. For the contact interfaces, 2D two-node surface/surface contact CONTAl71 element and the corresponding target segment TARGEl69 element are adopted for both electro-thermal analysis and thermal-mechanical analysis. This contact pair has degrees of freedom along the x and y direction, temperature, and potential, and therefore can simulate the mechanical, electrical, and thermal contact phenomena between contact interfaces.

#### 2.1.2. Boundary Condition

Figure 1b shows the schematic of boundary conditions. The reference temperature was set as 21 °C, and the convective heat transfer coefficient between air and cooling water was assumed as 19.4 Wm^−2^ K^−1^ and 1.5 × 10^4^ Wm^−2^ K^−1^, respectively.

#### 2.1.3. Material Properties and Welding Parameters

Temperature dependent material properties for the steel sheets, strips, and copper electrodes were used. The material properties of the electrode, steel, and strip are referred to in references [21,22], the detailed data of temperature-dependent thermo-physical properties of copper electrode, steel, and AISI304 steel strip are listed in Table A1, Table A2 and Table A3, respectively, in the Appendix, and the welding parameters are listed in Table 1.

#### 2.1.4. Contact Resistance Model

The contact resistance model employed in this study was a micro-electrical contact model proposed by Li [23] derived from Kohlrausch’s theory [24]. The voltage drop across the contact interface can be estimated by:

(1) $$V^{2}=4L(T_{S}^{2}-T_{0}^{2})$$

where *V* is the voltage drop across the contact interfaces, *T_S_* and *T*_0_ are the contact super temperature and the bulk temperature at the interfaces, respectively, and *L* is the Lorentz constant of iron (about 2.0 × 10^−8^(V/°C)^2^). In the present computations, *T_S_* at the sheet/sheet interface was specified to be the solidus of steel (1500 °C), and that at the electrode/sheet interface to be the melting point of the electrodes (1084 °C).

The temperature-dependent contact resistance of the interface can be obtained by dividing temperature-dependent voltage drop by weld current and the contact resistance value is then converted to an equivalent electrical resistivity assigned to the contact elements. This contact resistance model has been reported in references [25,26,27]; good agreements were reported between the calculated and experimental measurements in weld growth and dynamic resistance.

#### 2.1.5. Computational Procedure

**Figure 2:**
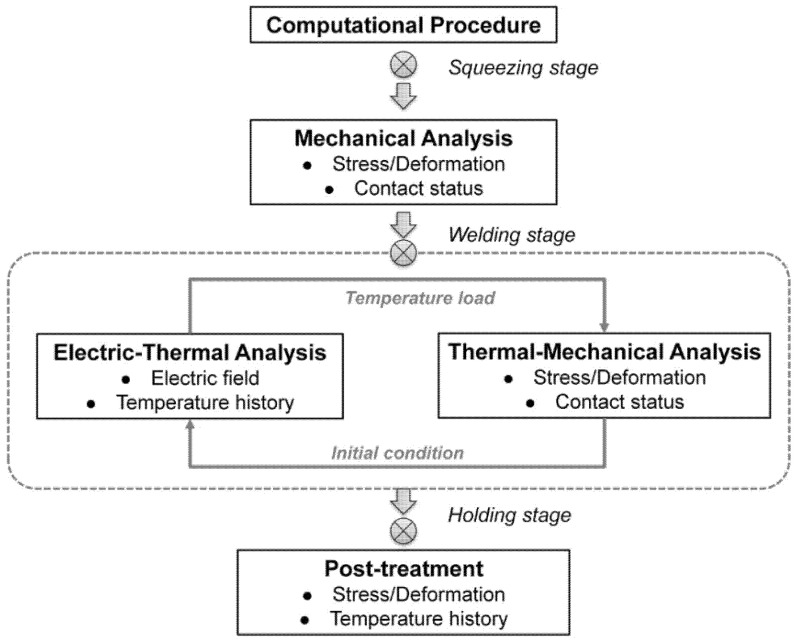
Schematic plot of FEM computational procedure.

The computational procedure is shown in Figure 2. At the squeezing stage, only mechanical analysis was conducted to calculate the contact status and stress distributions. Due to the difficulty of direct coupling of the electric-thermal-mechanical field, the coupled analysis of the welding stage was conducted in electric-thermal analysis and thermal-mechanical. The heat transfer result obtained from the electric-thermal analysis was applied as thermal load for the thermal-mechanical analysis, and the contact status of the thermal-mechanical was used as initial conditions for the next iteration of electric-thermal analysis. The electric-thermal analysis and thermal-mechanical iterated every 5 ms and the temperature of the contact elements was checked every 5 ms as well; once it exceeded the melting point, the corresponding contact element was disabled.

### 2.2. Temperature History

**Figure 3:**
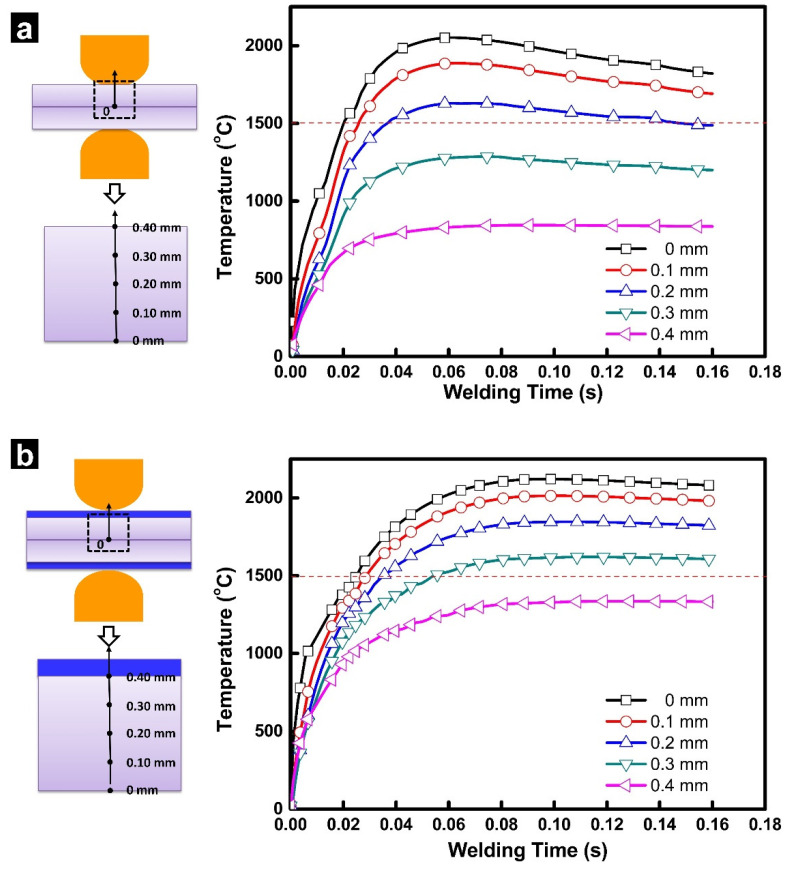
Calculated temperature histories in resistance spot welding of a 0.40 mm SAE1004 steel (results are presented for different levels of penetration in the steel sheet, ranging from 0 mm (faying interface) to 0.4 mm (electrode surface)), (**a**) without strip and (**b**) with a 0.10 mm AISI304 strip.

Figure 3 shows the transient temperature histories for the locations from the faying interfaces to the surface of the steel sheet estimated for RSW with/without inserted strips. As shown in Figure 3a, the temperatures in RSW of a 0.4 mm thick steel showed a “first-rise-then-fall” tendency, which were quite different from the monotonic increasing temperature history in resistance welding of thick steel (e.g., >1 mm) [28]. Referring to Figure 3a, the peak temperature of the weld zone exceeded the melting point of steel at 22 ms and reached the maximum (~2051 °C) at about 60 ms. After that, the temperature at the faying interfaces decreased in the rest of the welding process. At welding time of 160 ms, the temperature at the faying interface decreased to about 1820 °C. In this study, we mostly focused on the difference of the temperature history between RSW with and without inserted strips. The mechanism of the formation of the “first-rise-then-fall” temperature history in RSW of thin gage steels can be referred to reference [27].

As shown in Figure 3b, the calculated temperature history with inserted strips exhibits significant difference from traditional RSW. First, the peak temperature at the weld zone in RSW (2121 °C) with inserted strips is higher than that in traditional RSW (2051 °C). Moreover, with the inserted strips, the temperature at the faying interface did not show a significant decrease in the later part of the welding process. These two differences could be caused by the joule heat generation from the strips themselves and the additional contact resistance at sheet/strip interfaces.

### 2.3. Weld Formation Process

**Figure 4:**
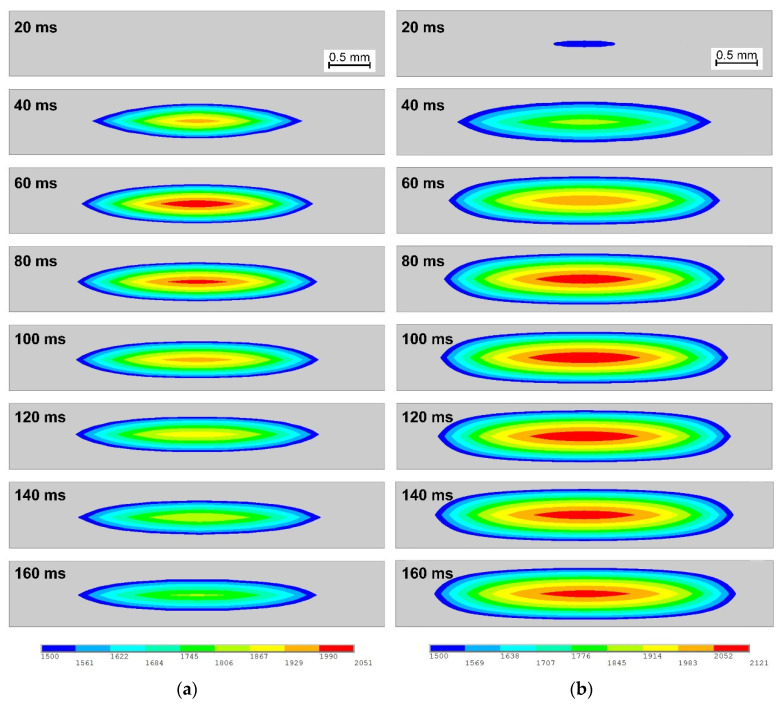
Comparison of weld formation process of (**a**) without strip and (**b**) with a 0.10 mm AISI304 strip.

Different temperature history would consequently lead to different weld initiation and growth process. Figure 4 shows the shape of the melting area of RSW with/without strips from 20 ms to 160 ms at interval of 20 ms. The gray area indicates the area with temperature lower than the melting point, while the colored contours show the area with temperature higher than melting point and thus equal to the shape of weld.

As shown in Figure 4a, the weld grew to 2.3 mm in diameter and 0.33 mm in depth after only 40 ms, and it kept growing to about 3.1 mm in diameter and 0.38 mm in diameter at 80 ms. However, the weld remained virtually unchanged from 80 ms to 160 ms. Referring to Figure 4b, the weld initiated within only 20 ms with inserted 0.10 mm thick AISI304 strip, earlier than in traditional RSW. Moreover, the weld increased considerably in both diameter and depth direction, and the shape of the weld changed. The calculated weld using inserted strip is about 3.8 mm in diameter and 0.63 mm. Compared to the weld produced by traditional RSW, the weld increased about 23% in diameter and 65% in depth by making use of the inserted strips. The more significant increase in weld depth could be related to the comparatively lower thermal conductivity of the strip material (i.e., stainless steel).

### 2.4. Electrode Surface Temperature

**Figure 5:**
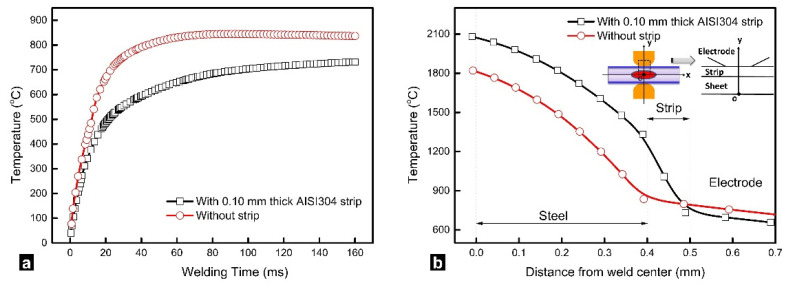
Comparison of (**a**) time-dependent electrode tip temperature and (**b**) temperature distribution of the symmetrical axis at the final moment of welding stage without strip and with a 0.10 mm AISI304 strip [4]. Reprinted with permission.

The time-dependent temperature at the electrode surface with and without strips during the welding process is presented in Figure 5a. As can be seen, the electrode surface temperature increased dramatically and reached its maximum (about 844 °C) and then slightly decreased in RSW without strips. By using inserted strips, the electrode surface temperature increased comparatively gradual, and a maximum electrode surface temperature (about 733 °C) was achieved at the end of the welding process. Figure 5b shows the temperature distribution along the *Y*-axis and explains the decrease in electrode surface temperature. A steep decrease in temperature can be observed where the strip is positioned. The heat transfer from the weld center to electrode was hindered by thermal resistance of the strip itself and strip/sheet, strip/electrode interfaces, which resulted in a lower electrode surface temperature.

### 2.5. Validation of Simulated Results

**Figure 6:**
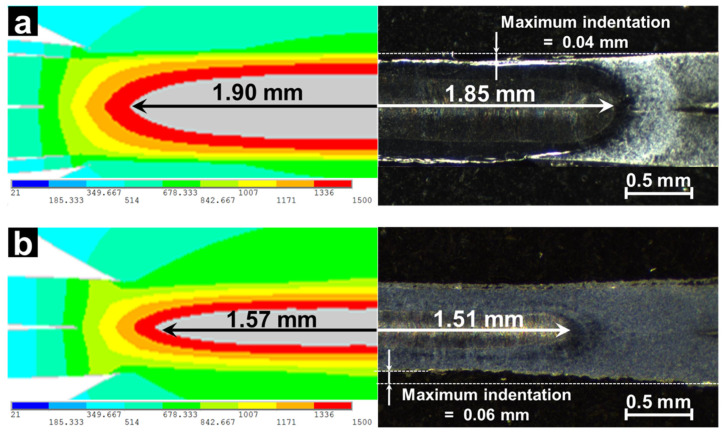
Comparison of calculated result and experimental result, (**a**) with a 0.10 mm AISI304 steel strip and (**b**) without strip [4]. Reprinted with permission.

Simulation results are compared to the experimentally tested ones. The equipment setup and testing procedures can be referred to reference [3]. As shown in Figure 6, the calculated size and shape of the weld nugget agreed well with the cross-sections of the welded sample produced using the same welding conditions as modeling, which suggested that the modeling results were qualified for the following study.

## 3. Results and Discussions

### 3.1. Development of Regression Model

#### 3.1.1. Second-Order Regression Equation

A second-order polynomial is used to build the relationship between the input variables and the response:

(2) $$y=a_{0}+\sum_{i=1}^{4}a_{i}x_{i}+\sum_{i=1}^{4}a_{ii}x_{i}{}^{2}+\sum_{i<j}\sum_{i=1}^{4}a_{ij}x_{i}x_{j}$$

where *y* is the response, *a*_0_ is the response of the central point, and *a_i_, a_ii_,* and *a_ij_* are regression coefficients of respective linear, squared, and interaction model terms.

#### 3.1.2. Design of Experiment

There are a number of parameters affecting the electrode tip temperature and weld size, and therefore we need to single out the most influential ones for the optimization. First, it has been reported that the material (i.e., thermal conductivity and electrical resistivity) and thickness of the strip played an important role on electrode tip temperature [3]. Considering that thermal and electrical conductivities of metals are proportional according to the Wiedemann–Franz Lorenz Law, they are treated as one factor in the optimization. Furthermore, previous studies [29] have pointed out that the welding current is a dominating process parameter influencing the electrode tip temperature and electrode life hence, the welding current should also be taken into account. Last, RSW with inserted strips is mostly applied for thin gage steel within the thickness range of 0.4–0.8 mm. Therefore, the input factors chosen in this study included the strip thickness, strip resistivity, welding current, and sheet thickness.

All these selected factors and their design levels are listed in Table 2. The top level was coded as +1, while the medium and bottom level were coded as 0 and −1, respectively. The electrode surface temperature, weld diameter, and maximum strip temperature were selected as responses, while the minimization of electrode tip temperature was the optimization objective, and the weld diameter and maximum strip temperature were utilized as the constraints. A 4-factor 3-level uniform design with 21 trials was selected. Furthermore, an additional trial was used as the central point. The design matrix of the variables and the calculated responses are tabulated in Table 3.

#### 3.1.3. Regression Models

According to Equation (2), there would be 14 terms (including linear, squared, and cross terms) for a full second order regression model of four factors. The analysis of variance (ANOVA) technique was further applied to test the statistical significance of the model terms and only the significant model terms would be reserved in the regression equation. The regression models of the three responses in terms of coded factors were demonstrated in Table 4. Detailed ANOVA results of the three models are listed in Table A4, Table A5 and Table A6 in the Appendix. The adequacy measures (i.e., R-squared, adjusted R-squared, F-value, and *p*-value) of the model are also labeled in Table 4. The closer R-squared is to 1, the more accurate the model is. The lower the *p* values, the higher the significance of the relating coefficient. As shown in Table 4, all three models had an R-squared value higher than 0.9 and a *p*-value less than 0.0001, suggesting the models are highly significant.

**Figure 7:**
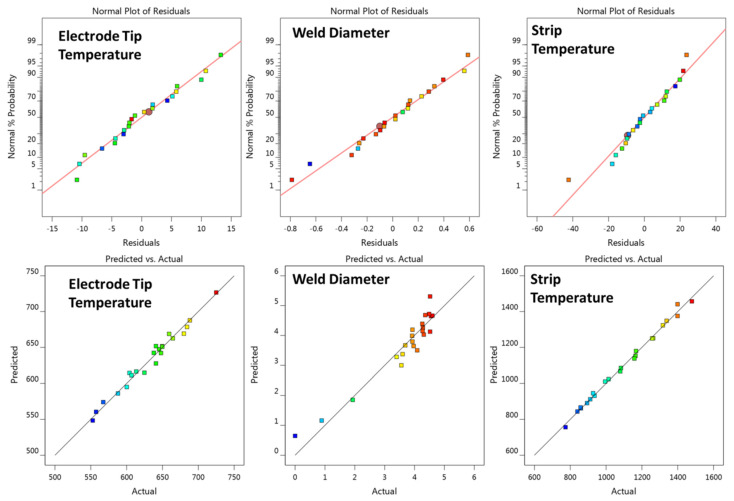
Normal probability plot of residuals and correlation plot of the three regression models.

Furthermore, Figure 7 shows the normal plot of residuals and the correlation plot of actual value and predicted value of the three regression models. All three normal plots of the residuals are approximately linear, indicating that the error terms are normally distributed. The three correlation plots are also approximately linear, supporting the condition that the predicted value agrees well with the actual value. 

### 3.2. Effect of Process Parameters on the Responses

#### 3.2.1. Electrode Tip Temperature

**Figure 8:**
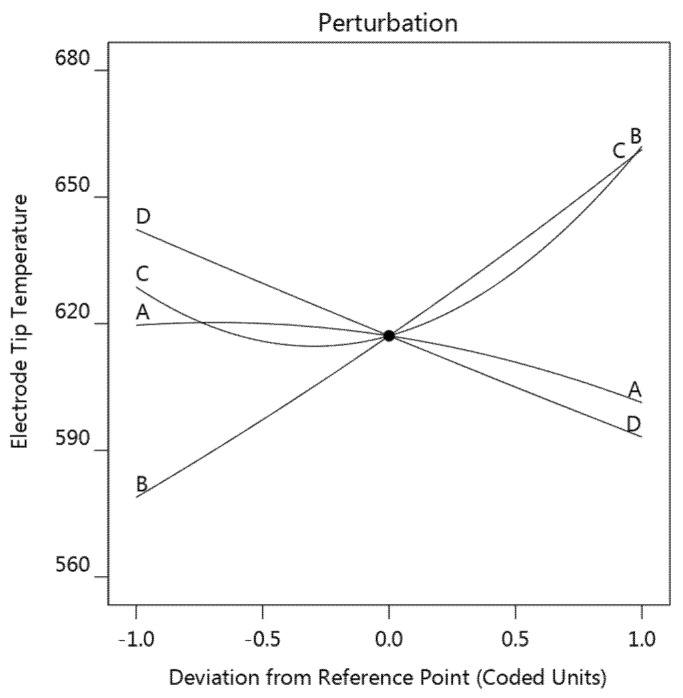
Perturbation plot showing the effect of the four factors on the electrode tip temperature (strip thickness (**A**), welding current (**B**), strip resistivity (**C**), and sheet thickness (**D**)).

A perturbation plot was employed to visually display the influence of the concerned parameters on the electrode tip temperature, as shown in Figure 8. This figure shows how the response varied with each factor while all other factors kept constant at 0 level. A steeper slope or curvature in a factor indicates that the response was more sensitive to that factor.

These four factors, in the order of decreasing influence on electrode tip temperature, were welding current B > sheet thickness D > strip resistivity C > strip thickness A. The welding current B showed the strongest positive effect on the electrode tip temperature, since the joule heat generation is proportional to the square of welding current, while it can be observed that both the sheet thickness D and strip thickness A have a negative effect on the electrode tip temperature. This result confirmed that the electrode tip temperature increased as the sheet became thinner. As for the strip resistivity, increasing of the strip resistivity resulted in a decrease of electrode tip temperature initially, the electrode tip temperature then started to rise as the strip resistivity exceeded −0.3 level. Generally, as the results indicated, it was not recommended that very high or very low strip resistivity be used.

**Figure 9:**
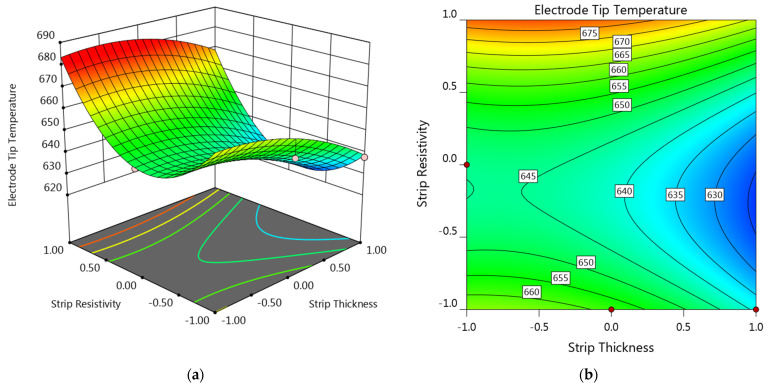
(**a**) Response surface and (**b**) contour plot showing the effect of factor A and C on the electrode tip temperature at B = 0 and D = −1.

Response surface and contour plot showing the strip thickness and strip resistivity on the electrode tip temperature are provided in Figure 9. The welding current was set as center level, while the sheet thickness was set as −1 level (equals to 0.4 mm). It can be seen that the electrode tip temperature at the left half was comparatively higher than the right half while the upper half was higher than the lower half. As shown in Figure 9, the electrode tip temperature was comparatively lower in the lower right section of the plots, which suggested that strips with comparatively higher thickness and lower resistivity would be more preferable for reducing the electrode tip temperature.

#### 3.2.2. Weld Diameter

**Figure 10:**
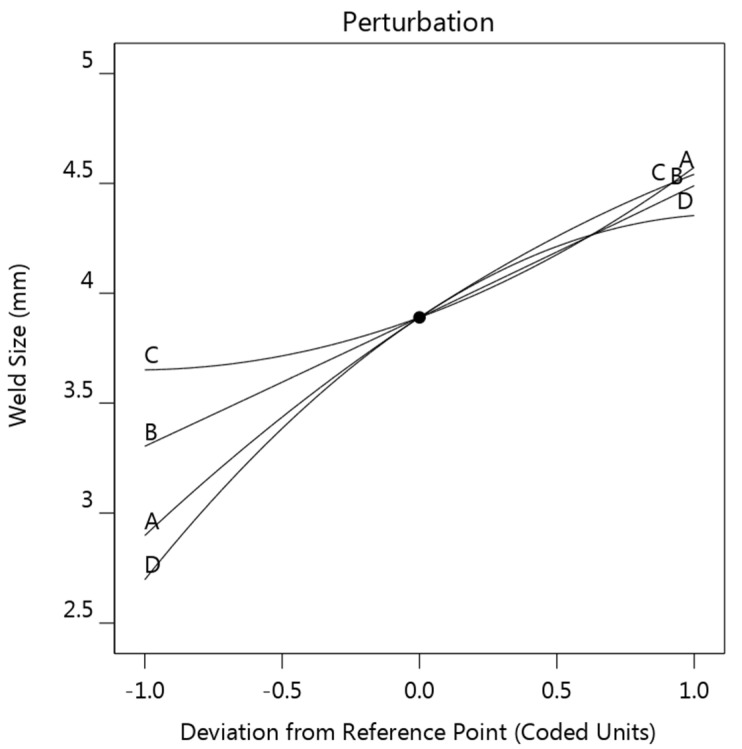
Perturbation plot showing the effect of the four factors on the weld diameter (strip thickness (**A**), welding current (**B**), strip resistivity (**C**), and sheet thickness (**D**)).

Figure 10 is the perturbation plot showing the influence of the four factors on the weld diameter. As can be seen, the effect of these four factors on the weld diameter was relatively balanced, and all factors showed a positive influence on the weld diameter. It was reasonable that increasing in all the four factors would promote the joule heat generation and lead to a greater weld diameter.

**Figure 11:**
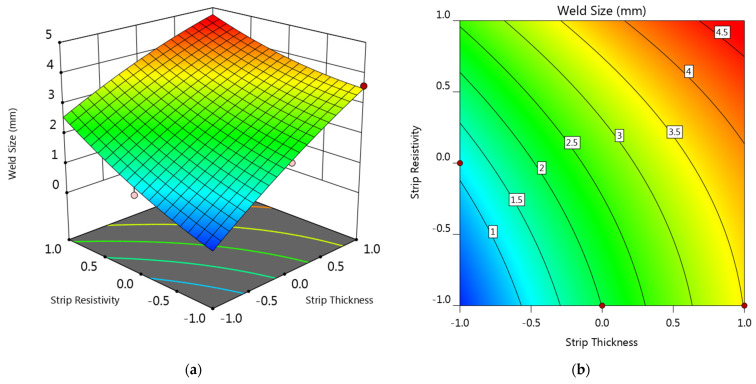
(**a**) Response surface and (**b**) contour plot showing the effect of factor A and C on the weld diameter at B = 0 and D = −1.

Response surface and contour plots of the interaction effects of strip thickness and strip resistivity on the weld diameter are provided in Figure 11. As can be seen, the smallest weld diameter occurred at (−1, −1), while the biggest weld diameter was obtained at (+1, +1). Overall, the response surface showed an increasing tendency along the diagonal from (−1, −1) to (+1, +1). Considering that the minimum acceptable weld diameter of 0.4 mm steel is 2.5 mm, it can be found that only the upper triangle part of the plot was qualified. This result suggested that a suitable strip should possess enough bulk resistance in order to maintain a qualified weld diameter. Strips with minus level of both strip thickness and strip resistivity were not qualified.

#### 3.2.3. Strip Temperature

**Figure 12:**
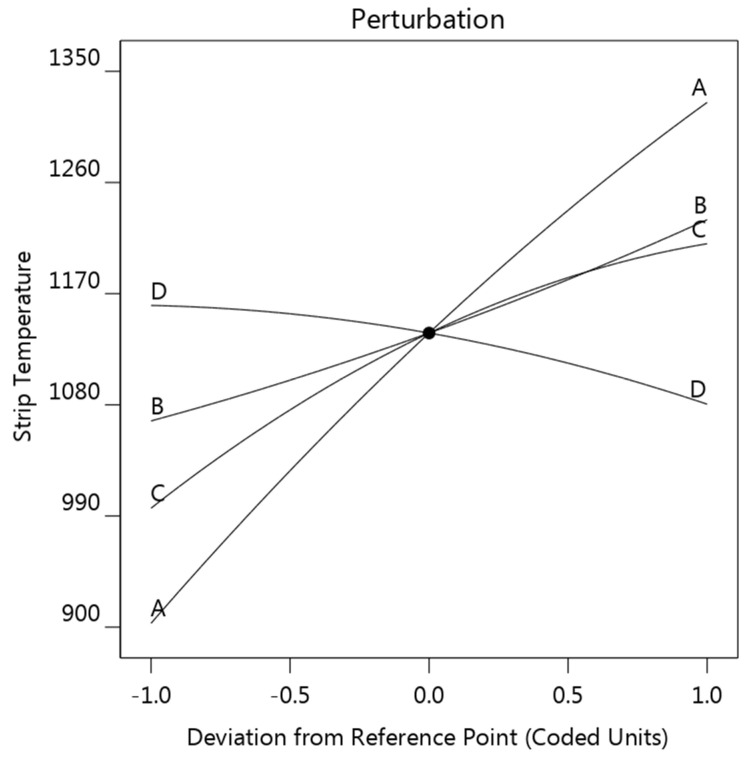
Perturbation plot showing the effect of the four factors on the strip temperature (strip thickness (**A**), welding current (**B**), strip resistivity (**C**), and sheet thickness (**D**)).

The strip temperature model was analyzed using a similar strategy; the perturbation plot is provided in Figure 12. As shown, the effect of each factor in strip temperature, sorting from most influential to least, is strip thickness A > strip resistivity C > welding current B > A sheet thickness D. Strip thickness, strip resistivity, and welding current exhibited a promoting effect on strip temperature, while the sheet thickness showed a slightly negative effect.

**Figure 13:**
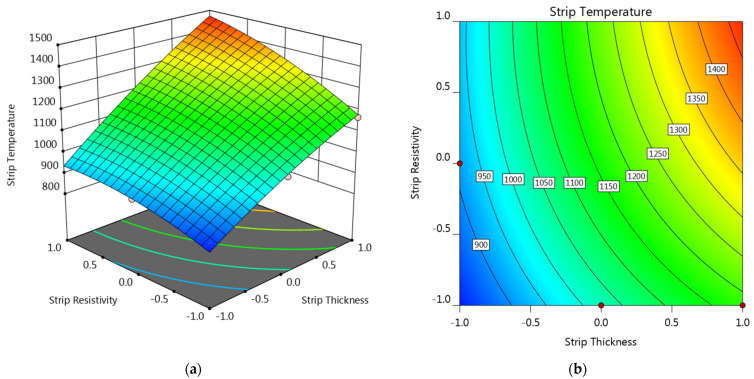
(**a**) Response surface and (**b**) contour plot showing the effect of factor A and C on the strip temperature at B = 0 and D = −1.

Figure 13 also shows the response surface and contour plot showing the strip thickness and strip resistivity on the strip temperature. The variation of strip temperature with strip resistivity and strip thickness was quite similar with that of weld diameter shown in Figure 11. A general increasing tendency can be observed along the diagonal from (−1, −1) to (+1, +1) of the response surface. However, the strip temperature, unlike the weld diameter, was not the bigger the better. Melting occurred at the sheet/strip interface should be avoided, thus the strip temperature should not exceed the melting point of both the steel sheet and the strip metal. From the consideration of strip temperature, strips with positive levels of both strips thickness and strip resistivity, the upper right section of the plot, would not be recommended.

### 3.3. Determination of the Process Window for a Preferable Strip

**Figure 14:**
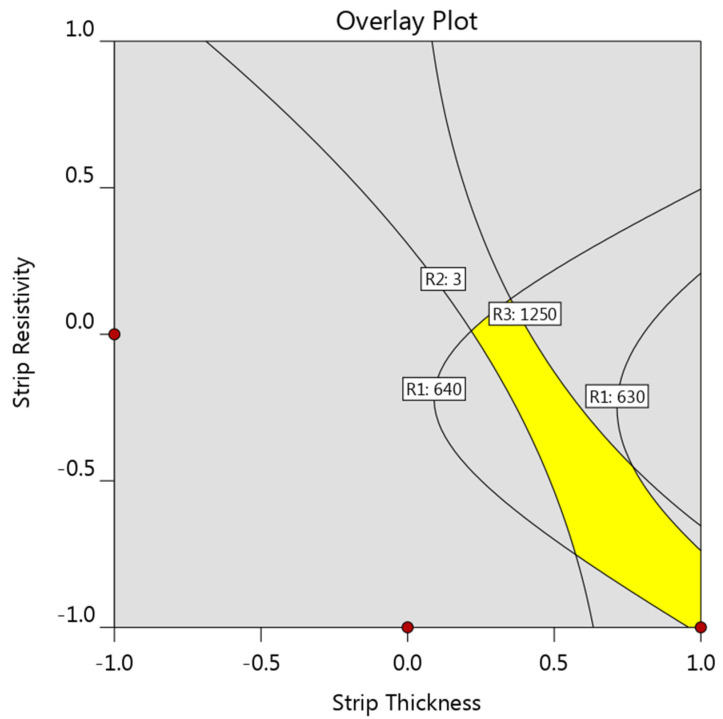
Overlay plot showing the preferable strip properties for a 0.40 mm thick steel.

Based on the aforementioned results, it can be seen that any improvement in one response usually resulted in deterioration of other responses. Hence, all three responses should be studied together. An optimization study is needed to find out the optimal strip to achieve a desirable electrode life and meanwhile a desirable weld diameter. Rather than identifying the very optimal strip, it would be of greater practical significance to figure out the process window for selecting a suitable strip for a specific steel sheet. Figure 14 shows the overlay plot of the feasible strip properties for a 0.4 mm thick steel sheet. The criteria for the three responses are that the electrode tip temperature should be lower than 640 °C, weld diameter bigger than the minimum acceptable one (i.e., 3.0 mm for a 0.4 mm thick steel sheet), and the strip temperature lower than 1250 °C. The areas highlighted in yellow on the overlay plots indicated the strip properties that met the aforementioned criteria.

### 3.4. Experiment Validation

To validate the developed models, confirmation experiments were carried out with strip properties chosen randomly from the graphical optimization results. An optimal strip material for a 0.4 mm steel sheet was selected randomly within the highlighted area determined as strip thickness of 0.40 and strip resistivity of −0.10 in coded level. By converting the coded level into actual strip properties, a Cu55Ni45 alloy strip with a thickness of 0.12 mm was selected.

The temperature developed at the electrode surface is difficult to measure experimentally. Fortunately, the electrode surface temperature can be reflected in the extent of electrode degradation, and the growth of the electrode tip diameter and the extent of surface alloying during electrode wear test are usually employed to evaluate the extent of electrode deterioration and therefore the electrode tip temperature. In order to validate the optimization results, experimental validation was conducted on a 0.4 mm SAE1004 steel.

**Figure 15:**
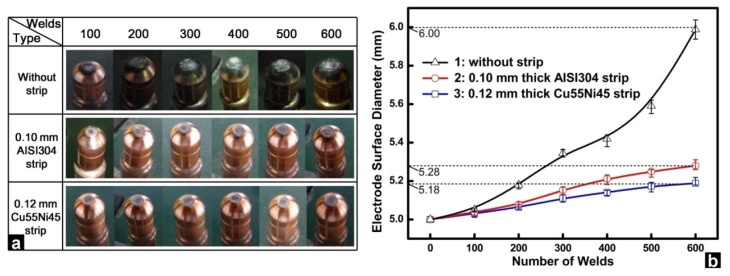
Comparison of (**a**) electrode surface profiles and (**b**) electrode surface diameter during electrode wear test [4]. Reprinted with permission.

Figure 15 shows the electrode profiles and the growth of electrode face diameters using different strips. In RSW without strips, a rough silvery layer could be observed on the electrode surface and the electrode surface was far smoother with inserted strips. Moreover, the growth rate of the electrode surface diameter was far slower by making use of inserted strips, especially the optimized 0.12 mm Cu55Ni44 strip.

**Figure 16:**
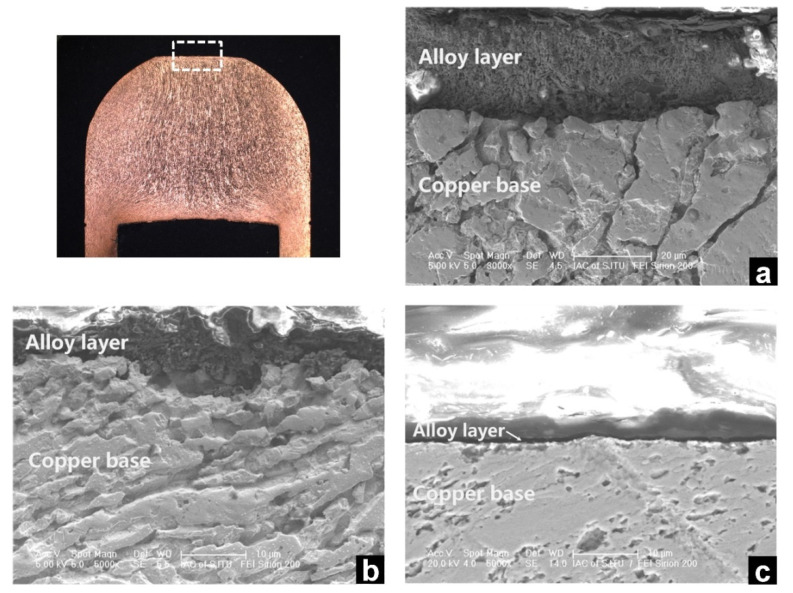
Cross-sections of the electrodes used after 600 welds, (**a**) no strip; (**b**) 0.10 mm AISI304 strip; and (**c**) optimized strip [4]. Reprinted with permission.

Figure 16 shows the cross-sections of the electrodes used after 600 welds. As shown in Figure 16, a thin layer of surface alloying could be observed on the three electrodes. The thickness of the thin layer differed widely. Without inserted strips, the thickness of the alloy layer was about 30 μm. It decreased to 6 μm by using 0.1 mm thick AISI304 strip and to 2 μm by using the optimal strip. The decrease in the thickness of the alloying layers implied a smaller extent of surface alloying occurring at the electrode surface and a lower temperature developed at the electrode surface, and the 0.12 mm Cu55Ni45 strip showed a better effect, which confirmed the validity of the optimization.

## 4. Conclusions

An investigation of resistance spot welding with inserted strips has been conducted using an integrated approach of FEM and RSM. The following points can be concluded:

(1) The inserted strips would lead to earlier weld initiation of weld and bigger final weld size in both diameter and thickness, and meanwhile lower electrode surface temperature.
(2) Strip thickness showed a negative effect on the electrode tip temperature, while the increase of strip resistivity led to a first-down-then-up electrode tip temperature. Both the strip thickness and the resistivity showed a positive effect on the weld diameter and the maximum strip temperature.
(3) A graphical optimization suggested a Cu55Ni45 strip with thickness of 0.12 mm for a 0.4 mm steel sheet.

## Figures and Tables

**Table 1:** Welding parameters.

Welding Parameters	Value
Electrode diameter (mm)	5
Electrode force (kN)	1.8
Welding current (kA)	5.7
Squeeze time (ms)	200
Weld time (ms)	160
Hold time (ms)	100

**Table 2:** Factors and the design levels.

Notation	Factor	Level		
		−1	0	1
A	Strip thickness (mm)	0.05	0.1	0.15
B	Weld current (kA)	5.0	5.5	6.0
C	Strip resistivity (μΩ·m)	0.3	0.55	0.8
D	Sheet thickness (mm)	0.4	0.6	0.8

**Table 3:** Design matrix and responses.

No.	Factors				Tip Temperature (°C)	Weld Diameter (mm)	Strip Temperature (°C)
	A	B	C	D			
1	0	1	1	1	684.3	4.55	1263
2	−1	0	0	−1	645.3	0.89	911.5
3	−1	0	1	0	659.3	3.93	935.5
4	1	0	1	0	640.9	4.52	1399
5	1	1	0	1	604	4.6	1338
6	1	−1	−1	0	587.9	3.92	1079
7	−1	0	−1	1	606.9	3.93	773.5
8	1	0	−1	−1	637.9	3.6	1167
9	−1	−1	0	−1	600.2	0	839.8
10	0	1	1	−1	725	4.31	1317
11	−1	−1	1	0	613.5	3.4	859
12	1	1	0	−1	664.4	4.49	1479
13	0	−1	1	−1	648	3.56	1164
14	−1	1	0	1	649.6	4.36	927.6
15	0	1	−1	0	679.9	4.28	1083
16	1	−1	0	0	567.4	4.26	1257
17	0	0	−1	−1	649.8	1.93	1014
18	0	−1	−1	1	557.4	3.69	857.7
19	0	−1	0	1	552.7	3.97	994.6
20	−1	1	−1	0	688.2	4.09	894
21	1	0	1	1	640.9	4.52	1399
22	0	0	0	0	624.8	4.27	1158

**Table 4:** Regression models of the three responses.

Response	R-Squared	Adj R-Squared	F-Value	*p*-Value	Response Equation
**Electrode Tip** **Temperature (°C)**	0.9816	0.9724	106.679	<0.0001	$\begin{array}{ll} Tip\;Temperature\; & =\;619.54-8.54A+42.32b+16.76C-24.11D\\ & -10.24AB-6.28A^{2}+27.60C^{2} \end{array}$
**Weld** **Diameter (mm)**	0.9187	0.8781	22.612	<0.0001	$\begin{array}{ll} Weld\;Diameter\; & =\;4.15+0.76A+0.51B+0.40C+0.74D\\ & -0.77AD-0.43CD-0.73D^{2} \end{array}$
**Strip** **Temperature (°C)**	0.9922	0.9882	253.237	<0.0001	$\begin{array}{ll} Strip\;Temperature\; & =\;1129.72+212.65A+84.38B+104.88C\\ & -37.88D+35.69AC-20.04A^{2}-31.07C^{2} \end{array}$

## Appendix A

Thermo-physical properties of the copper electrode, steel, and AISI304 stainless steel strip used in the finite element modeling are listed in Table A1, Table A2 and Table A3, respectively.

**Table A1:** Thermo-physical properties of the copper electrode.

Temperature (°C)	Young’s Modulus (GPa)	Electrical Resistivity (Ω·m × 10^−7^)	Specific Heat (J·kg^−1^·K^−1^)	Thermal Expansion Coefficient (K^−1^ × 10^−5^)
21	124	0.264	397.75	1.66
93	105	0.300	401.93	1.67
204	93	0.399	418.68	1.71
316	83	0.505	431.24	1.75
427	55	0.619	439.61	1.78
538	39	0.699	452.17	1.84
649	25	0.800	464.73	1.85
732	-	-	477.30	-
760	16	0.898	-	1.89
774	-	-	-	-
799	-	-	-	-
871	14	0.948	-	1.93
982	7	0.998	-	-

**Table A2:** Thermo-physical properties parameters of steel.

Temperature (°C)	Yield Strength (MPa)	Electrical Resistivity (Ω·m × 10 ^−7^)	Specific Heat (J·kg^−1^·K^−1^)	Thermal Expansion Coefficient (K^−1^ × 10^−5^)
21	188	1.42	443.8	1.1
93	178	1.86	452.2	1.15
204	-	2.67	510.8	1.22
316	140	3.76	564	1.3
427	122	4.95	611.3	1.35
538	-	6.48	661.5	1.4
649	75.8	8.18	762	1.46
732	-	-	1004.8	-
760	13.8	10.1	2386.5	1.4
774	-	11.2	1189.1	1.35
799	-	11.8	-	1.35
871	188	1.42	443.8	1.1
1093	178	1.86	452.2	1.15

**Table A3:** Thermo-physical properties of the AISI304 stainless steel.

Temperature (°C)	Young’s Modulus (GPa)	Electrical Resistivity (Ω·m × 10^−7^)	Specific Heat (J·kg^−1^·K^−1^)	Thermal Expansion Coefficient (K^−1^ × 10^−5^)
21	200	7.2	412	1.4
93	-	7.7	445	-
204	-	8.5	502	-
316	-	9.3	551	-
427	-	10.1	622	-
538	147	10.7	858	1.8
649	-	11.3	876	-
760	-	11.9	889	-
871	100	12.4	657	1.83
982	-	13.5	643	-
1093	50	15.0	690	1.86
1204	-	16.4	711	-
1755	15	-	-	1.9

ANOVA results for the regression model of electrode tip temperature, weld diameter, and strip temperature are listed in Table A4, Table A5 and Table A6, respectively.

**Table A4:** ANOVA analysis for the regression model of electrode tip temperature.

Source	Sum of Squares	df	Mean Square	F Value	Prob > F	
Model	39,900.97	7	5700.139	106.679	<0.0001	significant
A-A	1021.73	1	1021.726	19.122	0.0006	
B-B	24,934.92	1	24,934.923	466.660	<0.0001	
C-C	3595.16	1	3595.162	67.284	<0.0001	
D-D	7773.95	1	7773.949	145.490	<0.0001	
AB	719.82	1	719.820	13.472	0.0025	
A^2^	194.26	1	194.260	3.636	0.0773	
C^2^	3645.55	1	3645.548	68.227	<0.0001	
Residual	748.06	14	53.433			
Cor Total	40649.03	21				
R-Squared = 0.9816				Adj R-Squared = 0.9724		
Pred R-Squared = 0.9551				Adeq Precision = 40.2028		

**Table A5:** ANOVA analysis for the regression model of weld diameter.

Source	Sum of Squares	df	Mean Square	F Value	Prob > F	
Model	28.02	7	4.003	22.612	<0.0001	significant
A-A	7.33	1	7.326	41.386	<0.0001	
B-B	3.29	1	3.294	18.609	0.0007	
C-C	2.08	1	2.082	11.759	0.0041	
D-D	7.57	1	7.573	42.781	<0.0001	
AD	4.00	1	4.003	22.613	0.0003	
CD	1.25	1	1.250	7.060	0.0188	
D^2^	2.65	1	2.653	14.987	0.0017	
Residual	2.48	14	0.177			
Cor Total	30.50	21				
R-Squared = 0.9187				Adj R-Squared = 0.8781		
Pred R-Squared = 0.7943				Adeq Precision = 18.3722		

**Table A6:** ANOVA analysis for the regression model of strip temperature.

Source	Sum of Squares	df	Mean Square	F Value	Prob > F	
Model	926,739.32	7	132,391.332	253.237	<0.0001	significant
A-A	633,080.32	1	633,080.315	1210.953	<0.0001	
B-B	91,660.70	1	91,660.700	175.328	<0.0001	
C-C	153,993.21	1	153,993.206	294.557	<0.0001	
D-D	18,474.23	1	18,474.231	35.337	<0.0001	
AC	8660.82	1	8660.821	16.566	0.0011	
A^2^	2394.08	1	2394.079	4.579	0.0505	
C^2^	4758.75	1	4758.748	9.103	0.0092	
Residual	7319.13	14	522.795			
Cor Total	934,058.45	21				
R-Squared = 0.9922				Adj R-Squared = 0.9882		
Pred R-Squared = 0.9809				Adeq Precision = 49.7324		
